# Human enhancement: Genetic engineering and evolution

**DOI:** 10.1093/emph/eoz026

**Published:** 2019-09-28

**Authors:** Mara Almeida, Rui Diogo

**Affiliations:** 1 Centro de Filosofia das Ciências da Universidade de Lisboa, Faculdade de Ciências da Universidade de Lisboa, Campo Grande, 1749-016 Lisbon, Portugal; 2 Department of Anatomy, College Medicine, Howard University, 520 W St. NW, Numa Adams Building, Room 1101, Washington, DC 20059, USA

**Keywords:** enhancement, genetic engineering, genome editing, human evolution, evolutionary biology

## Abstract

Genetic engineering opens new possibilities for biomedical enhancement requiring ethical, societal and practical considerations to evaluate its implications for human biology, human evolution and our natural environment. In this Commentary, we consider human enhancement, and in particular, we explore genetic enhancement in an evolutionary context. In summarizing key open questions, we highlight the importance of acknowledging multiple effects (pleiotropy) and complex epigenetic interactions among genotype, phenotype and ecology, and the need to consider the unit of impact not only to the human body but also to human populations and their natural environment (systems biology). We also propose that a practicable distinction between ‘therapy’ and ‘enhancement’ may need to be drawn and effectively implemented in future regulations. Overall, we suggest that it is essential for ethical, philosophical and policy discussions on human enhancement to consider the empirical evidence provided by evolutionary biology, developmental biology and other disciplines.

Lay Summary: This Commentary explores genetic enhancement in an evolutionary context. We highlight the multiple effects associated with germline heritable genetic intervention, the need to consider the unit of impact to human populations and their natural environment, and propose that a practicable distinction between ‘therapy’ and ‘enhancement’ is needed.

## INTRODUCTION

There are countless examples where technology has contributed to ameliorate the lives of people by improving their inherent or acquired capabilities. For example, over time, there have been biomedical interventions attempting to restore functions that are deficient, such as vision, hearing or mobility. If we consider human vision, substantial advances started from the time spectacles were developed (possibly in the 13th century), continuing in the last few years, with researchers implanting artificial retinas to give blind patients partial sight [1–3]. Recently, scientists have also successfully linked the brain of a paralysed man to a computer chip, which helped restore partial movement of limbs previously non-responsive [4, 5]. In addition, synthetic blood substitutes have been created, which could be used in human patients in the future [6–8].

The progress being made by technology in a restorative and therapeutic context could in theory be applied in other contexts to treat non-pathological conditions. Many of the technologies and pharmaceutical products developed in a medical context to treat patients are already being used by humans to ‘enhance’ some aspect of their bodies, for example drugs to boost brain power, nutritional supplements, brain stimulating technologies to control mood or growth hormones for children of short stature. Assistive technology for disabled people, reproductive medicine and pharmacology, beside their therapeutic and restorative use, have a greater potential for human ‘enhancement’ than currently thought. There are also dual outcomes as some therapies can have effects that amount to an enhancement as for example, the artificial legs used by the South African sprinter Oscar Pistorius providing him with a competitive advantage.

This commentary will provide general ethical considerations on human enhancement, and within the several forms of so-called human biomedical enhancement, it will focus on genetic engineering, particularly on germline (heritable) genetic interventions and on the insights evolutionary biology can provide in rationalizing its likely impact. These insights are a subject often limited in discussions on genetic engineering and human enhancement in general, and its links to ethical, philosophical and policy discussions, in particular [9]. The rapid advances in genetic technology make this debate very topical. Moreover, genes are thought to play a very substantial role in biological evolution and development of the human species, thus making this a topic requiring due consideration. With this commentary, we explore how concepts based in evolutionary biology could contribute to better assess the implications of human germline modifications, assuming they were widely employed. We conclude our brief analysis by summarizing key issues requiring resolution and potential approaches to progress them. Overall, the aim is to contribute to the debate on human genetic enhancement by looking not only at the future, as it is so often done, but also at our evolutionary past.

## HUMAN ENHANCEMENT

The noun ‘enhancement’ comes from the verb ‘enhance’, meaning ‘to increase or improve’. The verb enhance can be traced back to the vulgar Latin *inaltiare* and late Latin *inaltare* (‘raise, exalt’), from ‘*altare*’ (‘make high’) and *altus* (‘high’), literally ‘grown tall’. For centuries human enhancement has populated our imagination outlined by stories ranging from the myths of supernormal strengths and eternal life to the superpowers illustrated by the 20th century comic books superheroes. The desire of overcoming normal human capacities and the transformation to an almost ‘perfect’ form has been part of the history of civilization, extending from arts and religion to philosophy. The goal of improving the human condition and health has always been a driver for innovation and biomedical developments.

In the broadest sense, the process of human enhancement can be considered as an improvement of the ‘limitations’ of a ‘natural version’ of the human species with respect to a specific reference in time, and to different environments, which can vary depending on factors such as, for example, climate change. The limitations of the human condition can be physical and/or mental/cognitive (e.g. vision, strength or memory). This poses relevant questions of what a real or perceived human limitation is in the environment and times in which we are living and how it can be shifted over time considering social norms and cultural values of modern societies. Besides, the impact that overcoming these limitations will have on us humans, and the environment, should also be considered. For example, if we boost the immune system of specific people, this may contribute to the development/evolution of more resistant viruses and bacteria or/and lead to new viruses and bacteria to emerge. In environmental terms, enhancing the longevity of humans could contribute to a massive increase in global population, creating additional pressures on ecosystems already under human pressure.

Two decades ago, the practices of human enhancement have been described as ‘biomedical interventions that are used to improve human form or functioning beyond what is necessary to restore or sustain health’ [10]. The range of these practices has now increased with technological development, and they are ‘any kind of genetic, biomedical, or pharmaceutical intervention aimed at improving human dispositions, capacities, or well-being, even if there is no pathology to be treated’ [11]. Practices of human enhancement could be visualized as upgrading a ‘system’, where interventions take place for a better performance of the original system. This is far from being a hypothetical situation. The rapid progress within the fields of nanotechnology, biotechnology, information technology and cognitive science has brought back discussions about the evolutionary trajectory of the human species by the promise of new applications which could provide abilities beyond current ones [12, 13]. If such a possibility was consciously embraced and actively pursued, technology could be expected to have a revolutionary interference with human life, not just helping humans in achieving general health and capabilities commensurate with our current ones but helping to overcome human limitations far beyond of what is currently possible for human beings. The emergence of new technologies has provided a broader range of potential human interventions and the possibility of transitioning from external changes to our bodies (e.g. external prosthesis) to internal ones, especially when considering genetic manipulation, whose changes can be permanent and transmissible.

The advocates of a far-reaching human enhancement have been referred to as ‘transhumanists’. In their vision, so far, humans have largely worked to control and shape their exterior environments (niche construction) but with new technologies (e.g. biotechnology, information technology and nanotechnology) they will soon be able to control and fundamentally change their own bodies. Supporters of these technologies agree with the possibility of a more radical interference in human life by using technology to overcome human limitations [14–16], that could allow us to live longer, healthier and even happier lives [17]. On the other side, and against this position, are the so-called ‘bioconservatives’, arguing for the conservation and protection of some kind of ‘human essence’, with the argument that it exists something intrinsically valuable in human life that should be preserved [18, 19].

There is an ongoing debate between transhumanists [20–22] and bioconservatives [18, 19, 23] on the ethical issues regarding the use of technologies in humans. The focus of this commentary is not centred on this debate, particularly because the discussion of these extreme, divergent positions is already very prominent in the public debate. In fact, it is interesting to notice that the ‘moderate’ discourses around this topic are much less known. In a more moderate view, perhaps one of the crucial questions to consider, independently of the moral views on human enhancement, is whether human enhancement (especially if considering germline heritable genetic interventions) is a necessary development, and represents an appropriate use of time, funding and resources compared to other pressing societal issues. It is crucial to build space for these more moderate, and perhaps less polarized voices, allowing the consideration of other positions and visions beyond those being more strongly projected so far.

Ethical and societal discussions on what constitutes human enhancement will be fundamental to support the development of policy frameworks and regulations on new technological developments. When considering the ethical implications of human enhancement that technology will be available to offer now and in the future, it could be useful to group the different kinds of human enhancements in the phenotypic and genetic categories: (i) strictly phenotypic intervention (e.g. ranging from infrared vision spectacles to exoskeletons and bionic limbs); (ii) somatic, non-heritable genetic intervention (e.g. editing of muscle cells for stronger muscles) and (iii) germline, heritable genetic intervention (e.g. editing of the C–C chemokine receptor type 5 (CCR5) gene in the Chinese baby twins, discussed later on). These categories of enhancement raise different considerations and concerns and currently present different levels of acceptance by our society. The degree of ethical, societal and environmental impacts is likely to be more limited for phenotypic interventions (i) but higher for genetic interventions (ii and iii), especially for the ones which are transmissible to future generations (iii).

## GENETIC ENGINEERING

The rapid advances in technology seen in the last decades, have raised the possibility of ‘radical enhancement’, defined by Nicholas Agar, ‘as the improvement of human attributes and abilities to levels that greatly exceed what is currently possible for human beings’ [24]. Genetic engineering offers the possibility of such an enhancement by providing humans a profound control over their own biology. Among other technologies, genetic engineering comprises genome editing (also called gene editing), a group of technologies with the ability to directly modify an organism’s DNA through a targeted intervention in the genome (e.g. insertion, deletion or replacement of specific genetic material) [25]. Genome editing is considered to achieve much greater precision than pre-existing forms of genetic engineering. It has been argued to be a revolutionary tool due to its efficiency, reducing cost and time. This technology is considered to have many applications for human health, in both preventing and tackling disease. Much of the ethical debate associated with this technology concerns the possible application of genome editing in the human germline, i.e. the genome that can be transmitted to following generations, be it from gametes, a fertilized egg or from first embryo divisions [26–28]. There has been concern as well as enthusiasm on the potential of the technology to modify human germline genome to provide us with traits considered positive or useful (e.g. muscle strength, memory and intelligence) in the current and future environments.

### Genetic engineering: therapy or enhancement and predictability of outcomes

To explore some of the possible implications of heritable interventions we will take as an example the editing (more specifically ‘deletion’ using CRISPR genome editing technology) of several base pairs of the CCR5 gene. Such intervention was practised in 2018 in two non-identical twin girls born in China. Loss of function mutations of the CCR5 had been previously shown to provide resistance to HIV. Therefore, the gene deletion would be expected to protect the twin baby girls from risk of transmission of HIV which could have occurred from their father (HIV-positive). However, the father had the infection kept under control and the titre of HIV virus was undetectable, which means that risk of transmission of HIV infection to the babies was negligible [29].

From an ethical ground, based on current acceptable practices, this case has been widely criticized by the scientific community beside being considered by many a case of human enhancement intervention rather than therapy [29, 30]. One of the questions this example helps illustrate is that the ethical boundary between a therapy that ‘corrects’ a disorder by restoring performance to a ‘normal’ scope, and an intervention that ‘enhances’ human ability outside the accepted ‘normal’ scope, is not always easy to draw. For the sake of argument, it could be assumed that therapy involves attempts to restore a certain condition of health, normality or sanity of the ‘natural’ condition of a specific individual. If we take this approach, the question is how health, normality and sanity, as well as natural per se, are defined, as the meaning of these concepts shift over time to accommodate social norms and cultural values of modern societies. It could be said that the difficulty of developing a conceptual distinction between therapy and enhancement has always been present. However, the potential significance of such distinction is only now, with the acceleration and impact of technological developments, becoming more evident.

Beyond ethical questions, a major problem of this intervention is that we do not (yet?) know exactly the totality of the effects that the artificial mutation of the CCR5 may have, at both the genetic and phenotypic levels. This is because we now know that, contrary to the idea of ‘one gene-one trait’ accepted some decades ago, a gene—or its absence—can affect numerous traits, many of them being apparently unrelated (a phenomenon also known as pleiotropy). That is, due to constrained developmental interactions, mechanisms and genetic networks, a change in a single gene can result in a cascade of multiple effects [31]. In the case of CCR5, we currently know that the mutation offers protection against HIV infection, and also seems to increase the risk of severe or fatal reactions to some infectious diseases, such as the influenza virus [32]. It has also been observed that among people with multiple sclerosis, the ones with CCR5 mutation are twice as likely to die early than are people without the mutation [33]. Some studies have also shown that defective CCR5 can have a positive effect in cognition to enhance learning and memory in mice [34]. However, it’s not clear if this effect would be translated into humans. The example serves to illustrate that, even if human enhancement with gene editing methods was considered ethically sound, assessing the totality of its implications on solid grounds may be difficult to achieve.

### Genetic engineering and human evolution: large-scale impacts

Beyond providing the opportunity of enhancing human capabilities in specific individuals, intervening in the germline is likely to have an impact on the evolutionary processes of the human species raising questions on the scale and type of impacts. In fact, the use of large-scale genetic engineering might exponentially increase the force of ‘niche construction’ in human evolution, and therefore raise ethical and practical questions never faced by our species before. It has been argued that natural selection is a mechanism of lesser importance in the case of current human evolution, as compared to other organisms, because of advances in medicine and healthcare [35]. According to such a view, among many others advances, natural selection has been conditioned by our ‘niche-construction’ ability to improve healthcare and access to clean water and food, thus changing the landscape of pressures that humans have been facing for survival. An underlying assumption or position of the current debate is that, within our human species, the force of natural selection became minimized and that we are somehow at the ‘end-point’ of our evolution [36]. If this premise holds true, one could argue that evolution is no longer a force in human history and hence that any human enhancement would not be substituting itself to human evolution as a key driver for future changes.

However, it is useful to remember that, as defined by Darwin in his book ‘On the Origin of the Species’, natural selection is a process in which organisms that *happen to be* ‘better’ adapted to a certain environment tend to have higher survival and/or reproductive rates than other organisms [37]. When comparing human evolution to human genetic enhancement, an acceptable position could be to consider ethically sound those interventions that could be replicated naturally by evolution, as in the case of the CCR5 gene. Even if this approach was taken, however, it is important to bear in mind that human evolution acts on human traits sometimes increasing and sometimes decreasing our biological fitness, in a constant evolutionary trade-off and in a contingent and/or neutral—in the sense of not ‘progressive’—process. In other worlds, differently from genetic human enhancement, natural selection *does not* ‘*aim*’ at improving human traits [38]. Human evolution and the so-called genetic human enhancement would seem therefore to involve different underlying processes, raising several questions regarding the implications and risks of the latter.

But using genetic engineering to treat humans has been proposed far beyond the therapeutic case or to introduce genetic modifications known to already occur in nature. In particular, when looking into the views expressed on the balance between human evolution and genetic engineering, some argue that it may be appropriate to use genetic interventions to go beyond what natural selection has contributed to our species when it comes to eradicate vulnerabilities [17]. Furthermore, when considering the environmental, ecological and social issues of contemporary times, some suggest that genetic technologies could be crucial tools to contribute to human survival and well-being [20–22]. The possible need to ‘engineer’ human traits to ensure our survival could include the ability to allow our species to adapt rapidly to the rate of environmental change caused by human activity, for which Darwinian evolution may be too slow [39]. Or, for instance, to support long-distance space travel by engineering resistance to radiation and osteoporosis, along with other conditions which would be highly advantageous in space [40].

When considering the ethical and societal merits of these propositions, it is useful to consider how proto-forms of enhancement has been approached by past human societies. In particular, it can be argued that humans have already employed—as part of our domestication/‘selective breeding’ of other animals—techniques of indirect manipulation of genomes on a relatively large scale over many millennia, albeit not on humans. The large-scale selective breeding of plants and animals over prehistoric and historic periods could be claimed to have already shaped some of our natural environment. Selective breeding has been used to obtain specific characteristics considered useful at a given time in plants and animals. Therefore, their evolutionary processes have been altered with the aim to produce lineages with advantageous traits, which contributed to the evolution of different domesticated species. However, differently from genetic engineering, domestication possesses inherent limitations in its ability to produce major transformations in the created lineages, in contrast with the many open possibilities provided by genetic engineering.

When considering the impact of genetic engineering on human evolution, one of questions to be considered concerns the effects, if any, that genetic technology could have on the genetic pool of the human population and any implication on its resilience to unforeseen circumstances. This underlines a relevant question associated with the difference between ‘health’ and biological fitness. For example, a certain group of animals can be more ‘healthy’—as domesticated dogs—but be less biologically ‘fit’ according to Darwin’s definition. Specifically, if such group of animals are less genetically diverse than their ancestors, they could be less ‘adaptable’ to environmental changes. Assuming that, the human germline modification is undertaken at a global scale, this could be expected to have an effect, on the distribution of genetically heritable traits on the human population over time. Considering that gene and trait distributions have been changing under the processes of evolution for billions of years, the impact on evolution will need to be assessed by analysing which genetic alterations have been eventually associated with specific changes within the recent evolutionary history of humans. On this front, a key study has analysed the implications of genetic engineering on the evolutionary biology of human populations, including the possibility of reducing human genetic diversity, for instance creating a ‘biological monoculture’ [41]. The study argued that genetic engineering will have an insignificant impact on human diversity, while it would likely safeguard the capacity of human populations to deal with disease and new environmental challenges and therefore, ensure the health and longevity of our species [41]. If the findings of this study were considered consistent with other knowledge and encompassing, the impact of human genetic enhancements on the human genetic pool and associated impacts could be considered secondary aspects. However, data available from studies on domestication strongly suggests that domestication of both animals and plans might lead to not only decreased genetic diversity per se, but even affect patterns of variation in gene expression throughout the genome and generally decreased gene expression diversity across species [42–44]. Given that, according to recent studies within the field of biological anthropology recent human evolution has been in fact a process of ‘self-domestication’ [45], one could argue that studies on domestication could contribute to understanding the impacts of genetic engineering.

Beyond such considerations, it is useful to reflect on the fact that human genetic enhancement could occur on different geographical scales, regardless of the specific environment and geological periods in which humans are living and much more rapidly than in the case of evolution, in which changes are very slow. If this was to occur routinely and on a large scale, the implications of the resulting radical and abrupt changes may be difficult to predict and its impacts difficult to manage. This is currently highlighted by results of epigenetics studies, and also of the microbiome and of the effects of pollutants in the environment and their cumulative effect on the development of human and non-human organisms alike. Increasingly new evidence indicates a greater interdependence between humans and their environments (including other microorganisms), indicating that modifying the environment can have direct and unpredictable consequences on humans as well. This highlight the need of a ‘systems level’ approach. An approach in which the ‘bounded body’ of the individual human as a basic unit of biological or social action would need to be questioned in favour of a more encompassing and holistic unit. In fact, within biology, there is a new field, Systems Biology, which stresses the need to understand the role that pleiotropy, and thus networks at multiple levels—e.g. genetic, cellular, among individuals and among different taxa—play within biological systems and their evolution [46]. Currently, much still needs to be understood about gene function, its role in human biological systems and the interaction between genes and external factors such as environment, diet and so on. In the future if we do choose to genetically enhance human traits to levels unlikely to be achieved by human evolution, it would be crucial to consider if and how our understanding of human evolution enable us to better understand the implications of genetic interventions.

## CONCLUSIONS

New forms of human enhancement are increasingly coming to play due to technological development. If phenotypic and somatic interventions for human enhancement pose already significant ethical and societal challenges, germline heritable genetic intervention, require much broader and complex considerations at the level of the individual, society and human species as a whole. Germline interventions associated with modern technologies are capable of much more rapid, large-scale impacts and seem capable of radically altering the balance of humans with the environment. We know now that beside the role genes play on biological evolution and development, genetic interventions can induce multiple effects (pleiotropy) and complex epigenetics interactions among genotype, phenotype and ecology of a certain environment. As a result of the rapidity and scale with which such impact could be realized, it is essential for ethical and societal debates, as well as underlying scientific studies, to consider the unit of impact not only to the human body but also to human populations and their natural environment (systems biology). An important practicable distinction between ‘therapy’ and ‘enhancement’ may need to be drawn and effectively implemented in future regulations, although a distinct line between the two may be difficult to draw.

In the future if we do choose to genetically enhance human traits to levels unlikely to be achieved by human evolution, it would be crucial to consider if and how our understanding of humans and other organisms, including domesticated ones, enable us to better understand the implications of genetic interventions. In particular, effective regulation of genetic engineering may need to be based on a deep knowledge of the exact links between phenotype and genotype, as well the interaction of the human species with the environment and *vice versa*.

For a broader and consistent debate, it will be essential for technological, philosophical, ethical and policy discussions on human enhancement to consider the empirical evidence provided by evolutionary biology, developmental biology and other disciplines.

## FUNDING

This work was supported by Fundação para a Ciência e a Tecnologia (FCT) of Portugal [CFCUL/FIL/00678/2019 to M.A.].

**Conflict of interest**: None declared.
